# Nitrogen use efficiency in cotton: Challenges and opportunities against environmental constraints

**DOI:** 10.3389/fpls.2022.970339

**Published:** 2022-08-22

**Authors:** Adnan Noor Shah, Talha Javed, Rajesh Kumar Singhal, Rubab Shabbir, Depeng Wang, Sadam Hussain, Hirdayesh Anuragi, Dinesh Jinger, Himanshu Pandey, Nader R. Abdelsalam, Rehab Y. Ghareeb, Mariusz Jaremko

**Affiliations:** ^1^Department of Agricultural Engineering, Khwaja Fareed University of Engineering and Information Technology, Rahim Yar Khan, Punjab, Pakistan; ^2^College of Agriculture, Fujian Agriculture and Forestry University, Fuzhou, China; ^3^ICAR-Indian Grassland and Fodder Research Institute, Jhansi, India; ^4^Department of Plant Breeding and Genetics, University of Agriculture, Faisalabad, Pakistan; ^5^College of Life Science, Linyi University , Linyi, Shandong, China; ^6^Department of Agronomy, University of Agriculture, Faisalabad, Pakistan; ^7^ICAR-Central Agroforestry Research Institute, Jhansi, Uttar Pradesh, India; ^8^ICAR-Indian Institute of Soil and Water Conservation, Research Centre, Anand, Gujarat, India; ^9^YSP UHF Nauni, Solan, Himachal Pradesh, India; ^10^Agricultural Botany Department, Faculty of Agriculture, Saba Basha, Alexandria University, Alexandria, Egypt; ^11^Plant Protection and Biomolecular Diagnosis Department, Arid Lands Cultivation Research Institute, City of Science Research and Technological Applications, Alexandria, Egypt; ^12^Smart Health Initiative and Red Sea Research Center, Division of Biological and Environmental Sciences and Engineering, King Abdullah University of Science and Technology, Thuwal, Saudi Arabia

**Keywords:** abiotic stresses, climate change, cotton, nitrogen use efficiency, physiological responses *GhNRT1.1*, *GhNRT1.2*, *GhNRT2.1*, *GhNRT2.2*

## Abstract

Nitrogen is a vital nutrient for agricultural, and a defieciency of it causes stagnate cotton growth and yield penalty. Farmers rely heavily on N over-application to boost cotton output, which can result in decreased lint yield, quality, and N use efficiency (NUE). Therefore, improving NUE in cotton is most crucial for reducing environmental nitrate pollution and increasing farm profitability. Well-defined management practices, such as the type of sources, N-rate, application time, application method, crop growth stages, and genotypes, have a notable impact on NUE. Different N formulations, such as slow and controlled released fertilizers, have been shown to improve N uptake and, NUE. Increasing N rates are said to boost cotton yield, although high rates may potentially impair the yield depending on the soil and environmental conditions. This study comprehensively reviews various factors including agronomic and environmental constraints that influence N uptake, transport, accumulation, and ultimately NUE in cotton. Furthermore, we explore several agronomic and molecular approaches to enhance efficiency for better N uptake and utilization in cotton. Finally, this objective of this review to highlight a comprehensive view on enhancement of NUE in cotton and could be useful for understanding the physiological, biochemical and molecular mechanism of N in cotton.

## Introduction

Cotton is a dual-purpose crop grown for fiber and oil purposes (Ali et al., 2019). Under field conditions, various factors such as environmental conditions and agricultural management practices significantly influenced cotton growth and productivity. Nitrogen is one of the most important limiting variables for cotton growth, productivity, and quality, and it is required more than other vital nutrients (Khan et al., 2017a,b,c). Normally, farmers rely on the excessive application of N to enhance growth and crop yield (Dong et al., 2012). Nevertheless, excessive rates of N not only outcomes in stagnant growth, prolonged crop maturity, and reduction in productivity, but also reduce NUE and environmental pollution (Khan et al., 2021; Niu et al., 2021; Huang et al., 2022; Javed et al., 2022a,b; Kumar et al., 2022; Wang et al., 2022).

Nitrogen, when applied with the appropriate method, time, and rate, significantly increased plant biomass production by improving the physiological events in plants including chlorophyll contents. However, to increase the cotton yield and profitability, farmers largely depend on the excessive application of N which can cause late crop maturity, decrease NUE and yield, and overall reduction in farm productivity (Devkota et al., 2013; Lehrsch et al., 2015). The previously published reports claimed that administering the appropriate N doses can increase biomass production and physiological characteristics which improve the yield and quality of cotton (Chen et al., 2016; Niu et al., 2021). Moreover, other published studies also suggested that reducing the rate of N with appropriate agronomic management operation not only improves the NUE but also increases the lint yield and quality (Dong et al., 2012; Luo et al., 2018). Besides, straw and straw-derived biochar incorporation into the soil is one of the major alternatives to prevent the further increase in synthetic nitrogen fertilizer during crop production (Chen et al., 2015; Xia et al., 2016). Nonetheless, the overuse of chemical N fertilizer in the cotton production system has not been ameliorated. Consequently, an in-depth understanding of decreasing N rates and their effect on NUE and crop performance is still crucial for cotton production. In addition to N rates, N forms also directly influence the uptake, accumulation, and distribution of N in cotton plants (Chen et al., 2018). According to Liu et al. (2021), N in the form of urea affects the root growth in cotton, which in turn, influence the N uptake and its accumulation. Similarly, N in the forms of controlled and slow-release fertilizers (such as polymer-based, urease, and nitrification inhibitors) was also reported to enhance N uptake and its accumulation in plants (Antille and Moody, 2021).

In addition to N fertilization, some other agronomic factors such as sowing dates, planting density, and tillage practices also influence the N uptake and NUE in cotton. Sowing time is the most important manageable factor in cotton as it is imperative in determining the lint yield and quality and N uptake in cotton (Khan et al., 2017a,b,c). Planting the crop at the optimum time is imperative not only for greater above and below-ground ground biomass but also for adequate uptake of N (Khan et al., 2017a,b,c). The plant density affects crop growth and development by inflicting interference among plants for space, light, nutrients, and moisture (Stephenson et al., 2011). Since nutrient uptake and plant density are linearly correlated, increasing plant population may lead to enhanced N uptake in reproductive organs. According to Jiang et al. (2013), high plant density imparts more N uptake and N translocation from vegetative parts to reproductive tissues. Furthermore, tillage also influences the mineralization and later release of nutrients from the soil (Khan et al., 2017a,b,c). Among commonly used tillage practices, zero tillage (ZT) retains more crop residues which leads to a reduction in soil erosion and evaporation and an increase in NUE compared to conventional tillage (CT; Lal et al., 2007). Also, it has been stated that ZT recorded more N uptake by cotton as compared to the CT.

In recent years, various molecular approaches are being discussed for enhancing N uptake and NUE in cotton crops. Among these, traditional phenotyping techniques are time-consuming, labor-intensive, expensive, and provide low-throughput and non-reliable phenotypes, impeding cotton breeding (Furbank and Tester, 2011). However, modern-day’s automated phenomic approaches including metabolic and transcriptomic approaches, are precise, time-saving, and high-throughput and can circumvent the limitations of conventional breeding (Singhal et al., 2021). These approaches are being utilized for the effective identification of high-yielding cultivars with additional superior qualities that might be used in the genetic modification of cotton. Likewsie, precision N use, N metabolism, growth attributes, N modelling, N-scheduling, high responsive genotypes and genes related to N uptake and transporters can help in improving NUE. A number of plant growth regulators such as auxin (root growth), cytokinin (delayed senescence), gibberellic acid (stem elongation), and ABA (stress tolerance) crucial for cotton growth and optimum use of these PGRs can enhance NUE and growth. Morover, the genes related to N metabolism, uptake and transport in cotton is crucial for targeting the several biosynthetic pathways, and could enhance the NUE (Yang et al., 2014; Sabagh et al., 2021). In this review-based study, we discussed different agronomic-and environmental-related factors affecting N uptake, its accumulation in plants, and ultimately NUE in cotton. Furthermore, we also discussed various agronomic and molecular approaches to enhance efficiency for more N uptake and its utilization in cotton. The mechanism of N uptake, transport and accumulation in cotton further help in balanced N management and improving NUE.

## N accumulation in cotton: Factors affecting N accumulation

### N forms

Nitrogen accumulation in cotton crops is heavily dependent on crop growth phase, N application rates, N sources, plant species, and soil and environmental conditions (Yang et al., 2013). Nitrogen forms directly influence the accumulation and distribution of N in cotton plants, by affecting the root metabolism (Chen et al., 2018). For example, a recent study by Liu et al. (2021) stated that N when applied in the form of urea affects the root growth in cotton, which in turn, influence the N uptake and its accumulation. Similarly, N application in the forms of controlled and slow-release fertilizers such as polymer-based, urease, and nitrification inhibitors also reported enhancing N uptake and its accumulation in plants (Antille and Moody, 2021). Nitrogen supply from enhanced-efficiency nitrogenous fertilizers, such as 3,4-Dimethylpyrazole phosphate, has been well recognized to reduce N losses and enhance its efficiency in crops including cotton (Suter et al., 2020; de Paulo et al., 2021; Qiao et al., 2021). Recently, nano-fertilizers with unique nano properties (1–100 nm size of the particle) have gained popularity due to their higher nutrient use efficiency (Suppan, 2017). Sohair et al. (2018) reported the enhanced yield, yield components, and fiber properties of Egyptian cotton by nano-fertilizer applications.

### N quantity

N application rates also influence N uptake and its accumulation in plants. It is generally reported that increased N rates can increase the productivity of the cotton crop. For cotton crops, N application between 0 and 600 kg ha^−1^ is reported to positively influence the N accumulation, and reduce leaching and runoff losses (Yang et al., 2013). Normally, the cotton crop grows well when N is applied in the range of 50–412 kg ha^−1^ (Zurweller et al., 2019). In a recent field study, Wang et al. (2021a,b,c,d) recorded the highest N accumulation in cotton plants when N was applied at 300 kg ha^−1^. Furthermore, Hou et al. (2021) recorded the highest nitrogen accumulation in cotton when N was applied at 223.88 and 242.77 kg ha^−1^. However, excessive application of N causes soil and environmental pollution, degrading soil biological activities, nutrient imbalance, impairs growth, and ultimately crop productivity in terms of reduced yield and NUE (Ju et al., 2009; Liu et al., 2013; Chen et al., 2021). According to Macdonald et al. (2016), a high dose of N also causes leaching and runoff losses owing to reducing the availability of N in the rhizosphere. In addition, a deficiency of N reduced root growth (Chen et al., 2021). The root system in cotton directly influences the N use efficiency, which can be increased by regulating the root system (Chen et al., 2018, 2021). Nitrogen stress in cotton reduced the root growth by regulating cell wall growth, reducing cellular polysaccharide and glucan levels, increasing lignin contents, and expression of various genes involved in cell wall components such as *EXPA17, EXLA1, LAC4*, and *NAC081* (Chen et al., 2021). Nitrogen application rates significantly influence the root structure, and moderate N application rates increase cotton root length and promote coordinates of the root-shoot relationship (Chen et al., 2020). Nitrogen stress also inhibits the carriage of auxin and increases lateral root elongation (Krouk et al., 2010). The change in the level of abscisic acid (ABA) and salicylic acid (SA) in lateral roots under varying N application rates were reported by Chen et al. (2021). Also, SA suppresses root development while promoting the growth of lateral plant roots and root primordium (Chen et al., 2021). Reduced root length in cotton was also associated with the activities of SA, JA, and ABA.

### Accumulated period

In cotton, maximum N uptake largely occurred during 49–71 days after sowing, and the most quantity of N is accumulated during late growth stages, i.e., flowering and boll setting stages (Boquet and Breitenbeck, 2000; Yang et al., 2013). However, published reports have reported variable periods for N absorption in cotton crops. For example, Zhao et al. (2010) stated that cotton crop uptake maximum N between the period of 54–110 days after planting. Similarly, according to Wang et al. (2003), maximum N absorption takes place 90 days after planting. It is well stated that late N application is more beneficial to increase NUE and more N accumulation at the fruit developing stage in cotton crops (Tang et al., 2012). Basal N application in cotton is not recommended because this method losses 80% of applied N under field conditions, as compared to topdressing in which only 40% of losses are documented (Yang et al., 2013). In the same study, Yang et al. (2013) reported that maximum N accumulated at the reproductive stage in cotton because seeds are a major constituent of N than other organs. Recently, Che et al. (2021) recorded the highest N uptake and its accumulation when N was applied at 375 kg ha^−1^ at the flower boll stage in cotton under full irrigation supply.

### N distribution in crop plant

**Figure 1:**
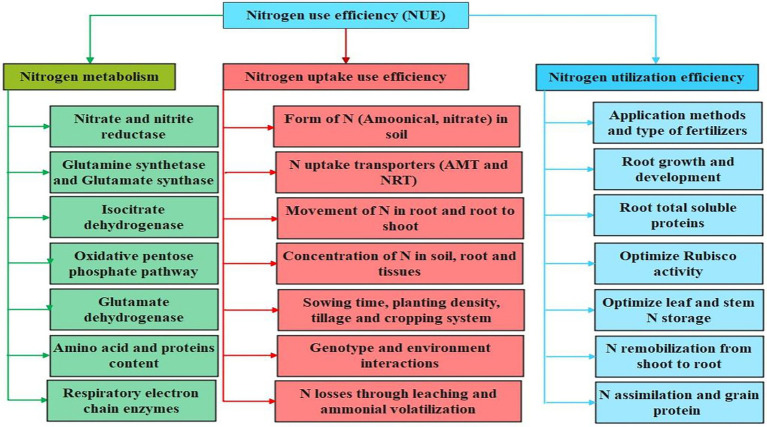
Major components involved in nitrogen use efficiency (NUE) in cotton plant. The nitrogen metabolism, uptake and utilization are crucial mechanisms involved in enhancing NUE in cotton. The activation of N transporters, plant growth indices, and N metabolizing pathways involved in facilitating N metabolism, uptake and utilization are highlighted.

N application rates and time of application, soil and environmental conditions, crop species, and other management practices influenced the N distribution in different plant organs. Plant roots play an essential role in N uptake and its transport (Bertoni, 2012). Different N forms and concentrations, soil properties, and root architecture can influence the N transport into the plant system (Khan et al., 2017a,b,c). Roots, which consume energy, mainly uptake N in the form of nitrate which is among the major forms of N present in the soil (Hu et al., 2014). Plants can uptake nitrate mainly through two systems/infinities: low-and high-affinity transporter systems by involving different transporter proteins such as nitrate transporters (NRT1 and NRT2) and peptides transporter proteins (Nacry et al., 2013; Wang et al., 2018). These proteins are activated through various genes such as putative NRT1/PTR genes including NRT1.4 and AtNRT1:4 in model Arabidopsis plants and different NPF genes in crop plants (Chiu et al., 2004; Tsay et al., 2007; Von Wittgenstein et al., 2014). Following uptake by plant roots, NO_3_-is reduced to NO_2_-by the nitrate reductase (NR) enzyme, and this NO_2_-is further reduced to NH_4_^+^ by the glutamate synthetase (GS) enzyme (North et al., 2009; Mokhele et al., 2012; Wang et al., 2018). Ammonia, plays an essential role in nitrogen metabolism, assimilated in cotton by the negotiation of different enzymes such as glutamine synthetase and glutamine oxoglutarate aminotransferase (Khan et al., 2017a,b,c). Most soil uptake nitrogen is assembled in vacuoles where it is reserved as an osmotic ion and then this nitrogen is let out in the cytosol for reduction. During the process of photorespiration in plants, nitrogen, in the form of glycine, is distributed for re-assimilation. At the reproductive stage, leaves can remobilize the nitrogen and transfer it to the reproductive plant parts through the hydrolysis of nitrogenous compounds including proteins (Khan et al., 2017a,b,c). The process of nitrogen metabolism is take place through different enzymes such as nitrate reductase (NR), nitrite reductase, and glutamate synthase (Ahmad and Abdin, 1999; Mokhele et al., 2012). Various transporters including *AtNRT1.5* and *AtNRT1.8* actively participated during the loading and unloading of nitrate from shoot vasculature/ root stele (Lin et al., 2008). According to Wang and Tsay (2011), another transporter *AtNRT1.9* is involved during the loading of nitrate ions into the root phloem and facilitates in downward transport of nitrate in roots. The components, i.e., N metabolism, utilization, and uptake, related to NUE in cotton plants are schematically highlighted in Figure 1.

## Factors related to N uptake

### Agronomic factors

#### Sowing time

Sowing time is considered to be the most important manageable factor in the cotton crop. It is an imperative factor in determining the yield and quality of lint as well as N uptake in cotton crops. Planting the crop at the optimal period is a must for higher above-and below-ground growth and appropriate N uptake. Sowing time greatly affects N status in cotton leaves, which is positively correlated with canopy photosynthetic ability (Khan et al., 2017a,b,c). Synchronization of crop N demands with its supply is significant for enhancing crop NUE. The requirement of N for a crop is largely associated to yield potential, which in turn is related to N supply during sowing time as well as later stages of crop growth (Yousaf et al., 2016). It has been reported that higher lint yield owing to early sowing was mainly attributed to adequate N uptake led to better boll formation, similarly, lint yield declined with late sowing due to the less time available to mature bolls (Bange et al., 2008). Sowing date also influences the availability, accumulation, distribution, and utilization of N (Yadvinder-Singh et al., 2005). Venugopalan et al. (2012) reported that N uptake and utilization efficiency were reduced in late sowing over the normal planting time of the Bt cotton.

#### Planting density

The plant density affects crop growth and development by inflicting interference among plants for space, light, nutrients, and moisture (Stephenson et al., 2011). Since nutrient uptake and plant density are linearly correlated, increasing plant population may lead to enhanced N uptake in reproductive organs. High plant density imparts more N uptake and N translocation from vegetative parts to reproductive tissues (Jiang et al., 2013). It has been reported that cotton planted at 15 cm plant spacing resulted in higher N uptake over cotton planted at 22, 30, 37, and 45 cm spacing in the Multan region of Pakistan (Zaman et al., 2021). Similarly, Panhwar et al. (2018) also reported that close plant spacing in cotton led to higher leaf area, lint yield, chlorophyll contents, and N uptake. Further, investigations revealed that the highest N contents in cotton were recorded under high-density planting (12 plants/m^2^) and minimum N content was found under low-density planting (8 plants/m^2^) in the Wuhan region of China (Shah et al., 2021a,b). Plants with higher plant density benefited from enhanced N uptake during initial to peak flowering. Despite an accelerated rate of nutrient accumulation per unit area, plants with higher plant density might have accumulated lesser nutrients on a per plant basis (Xue et al., 2013). The N recovery in cotton was recorded the highest with narrow plant spacing as compared to wider plant spacing. This might be due to more exploration of soil under the close row and plant spacing (Venugopalan and Blaise, 2001).

#### Fertilization

Cotton is a long-duration crop that requires an adequate quantity of nutrients to be uptaken for successful growth and yield. The application of nutrients also not only affects the yield of cotton but also the uptake of N and NUE (Prasad, 2019). It has been reported that plant N uptake over the entire growth period of cotton improved with the increasing N application dose. However, agronomic efficiency and recovery efficiency of N declined with the increasing N application dose (Pengcheng et al., 2017). In Syria, it has been observed that maximum N uptake (354 kg N ha^−1^) in cotton was recorded with the application of 200 kg N ha^−1^. Further increase in the application dose of N diminished the N uptake of cotton, suggesting that increasing N input up to 200 kg N ha^−1^ led to an increase in N uptake (Janat, 2008). In China also the total N uptake in cotton increased with the N application rates. The total N uptake in N-applied cotton was about 46% greater than the cotton without N application (Chen et al., 2010). Similarly, in the Punjab region of Pakistan, application of N at 180 kg N ha^−1^ showed higher uptake of N in cotton as compared to 120 kg N ha^−1^ (Shah et al., 2021a,b).

#### Plant growth regulator

It has been reported that supplementation of plant growth regulators in cotton crops improves the antioxidant defense systems, water use efficiency, nutrient availability, and uptake (Noreen et al., 2020). The application of humic acid imparts in releasing blocked or occluded phosphorus from soil collides and thus, increases its availability which improves N uptake and utilization, ultimately enhancing cotton yield (Khan et al., 2017a,b,c). Likewise, K^+^ content in cotton leaf and root increased by 20%–34% and 2%–8% respectively, due to the application of sodium nitroprusside at 300 μM (Kong et al., 2016). Application of salicylic acid at 100 μM to cotton seedlings for 3 days significantly improved Cu accumulation in roots, while reducing its translocation to aboveground parts (Mei et al., 2015). Supplementation of Brassinosteroids or putrescine to cotton plants resulted in enhanced uptake of N, P, and K compared to untreated plants (Ahmed et al., 2016). The application of Mepiquat chloride and Miantaijin increased the lint yield and K uptake in cotton in China. The plant growth regulators also improved the partial factor productivity, agronomic efficiency, and apparent recovery efficiency of K fertilizer (Yang et al., 2014).

#### Tillage

Tillage is an important practice to obtain a good crop yield from agricultural land. Crop productivity depends on the nutrient applied or present in the soil. Tillage influences the mineralization and later release of nutrients from the soil. It has been demonstrated that zero tillage (ZT) retains crop residues which leads to a reduction in soil erosion and evaporation and an increase in NUE compared to conventional tillage (CT; Lal et al., 2007). It has been revealed that uptake of N by cotton was recorded significantly highest in ZT as compared to the CT. The adequate moisture content in ZT helped to restore high N in soil which led to improved uptake by cotton (Khan et al., 2021). Similarly, the highest uptake of N, P, and K in cotton was recorded in ZT as compared to CT in the Akola region of Maharashtra (Wagh et al., 2016). ZT can also influence the amounts of supplemental N needed to secure optimal cotton yields. Bronson et al. (2001) reported that 20–35 kg ha^−1^ additional N was required under ZT than with CT. It has been well explained that tillage incorporates crop residue and increases soil aeration, which serves to speed up the decomposition of both residue and existing organic matter and thereby enhance the release of N (Boquet et al., 2004). In the Nagpur region of Maharashtra cotton yield and partial factor productivity improved significantly due to the adoption of ZT. This was mainly due to a better soil hydro-thermal regime and improved weed control (Blaise, 2006).

#### Cropping system

A sustainable cropping system always maintains a balance between the input and output of N by imparting adequate uptake and a minimum loss from soil (Herridge et al., 2008). Investigations from the 2 years rotation of cotton–soybean resulted in higher productivity and NUE as compared to the cultivation of sole cotton (Blaise, 2011). The introduction of short-duration wheat varieties resulted in an intensification of the cotton-wheat cropping system by including legume crops in the rotation. Legumes can fix atmospheric N in considerable amounts in their root nodules and the integration of legumes in the existing cropping systems could minimize the N requirement of the system (Blaise, 2017). It has been demonstrated that legumes could facilitate the growth and productivity of non-legume crops through nutrient sharing as legumes not only fix atmospheric N for their consumption (Sitienei et al., 2017) but also for crops grown in succession (Hu et al., 2017). It has been reported that intercropping of cotton with mungbean enhanced the total N uptake, NUE, and PFP for N by 28%–45%, 27%–44%, and 16%–19% as compared to sole planting of cotton in northwest China (Liang et al., 2020). In the Indo-Gangetic plains of India intercropping of cowpea with cotton had higher production efficiency, N uptake, and NUE compared to sole cotton (Rajpoot et al., 2014).

### Abiotic factors

#### Soil salinity

Soil salinity is one of the abiotic factors affecting the growth, development, and photosynthesis in plants and is greatly correlated with osmotic stress, nutritional imbalance, and specific ion toxicity (Nazar et al., 2011). Nutrient uptake is drastically reduced under soil salinity stress, and it may inhibit nutrient translocation and eventually lead to a yield penalty (Pessarakli, 2001). It is well known that sodic or alkali soil contains huge amounts of carbonates and bicarbonates of Na^+^. It has been demonstrated that growing cotton in sodic soils resulted in less dry matter production as compared to cotton growing in non-sodic soils (Dodd et al., 2010). Inhibition of nutrient uptake and growth of cotton in sodic soils are largely related to the poor physical condition or soil structure of these soils, due to the huge content of Na^+^ in the soil solution (Dodd et al., 2010). Soil salinity is the biggest hurdle for seed germination and crop emergence. Cultivation of cotton in saline soils leads to reduced leaf area and poor seed germination (Dong, 2012). It has been reported that application of silicon fertilizer application could mitigate soil salinity stress and improve crop yield (Jinger et al., 2020).

#### Drought

Failure of monsoon or paucity of water leads to drought stress which affects the growth and yield of the crops (Ahmad et al., 2021; Ali et al., 2021; Bahar et al., 2021; Chowdhary et al., 2021; Islam et al., 2021; Mahmood et al., 2021; Ahmad et al., 2022a,b; Akhtar et al., 2022). Reduced availability of moisture during the crop growing season is the major factor responsible for poor crop productivity (Noman et al., 2017). Photosynthetic and respiratory activities of crops are severely inhibited due to drought stress (Alghabari et al., 2016). Drought stress also plays a pivotal role in the poor mineral nutrition of plants since most of the plant nutrients are translocated along with water (Khawar et al., 2016). In open-field situations, the application of nutrients during drought conditions has no impact on crop growth since nutrients need adequate soil moisture to improve yield. A low concentration of K^+^ ions directly causes membrane damage and deformation of ionic homeostasis. Additionally, moisture scarcity affects the physiology of nutrients in plants and can retard the activity of nitrate reductase and glutamine synthetase associated with the assimilation of NH_4_^+^ to proteins (Rizhysky et al., 2004). In general, N uptake is improved, P uptake is reduced, and K remains unaffected during drought stress. Nevertheless, nutrient relations turn out to be more convoluted owing to interaction effects among different nutrients (Fahad et al., 2017).

#### Waterlogging

Excess moisture also arrests the nutrient absorption, photosynthesis, growth, and reproductive development of cotton crops which ultimately results in loss of crop yield (Najeeb et al., 2015a). Waterlogging creates an anoxia situation that could be one of the imperative aspects accountable for yield penalty in cotton due to the production of ethylene in plant tissue under excess water conditions (Najeeb et al., 2015b). Inadequacy of particular nutrients in cotton leaves could lead to a decline in chlorophyll and photosynthetic activity. The application of particular nutrients could enhance photosynthesis and chlorophyll content (Ashraf et al., 2011). On the contrary, Zhou and Oosterhuis (2012) reported that the application of specific nutrients could not improve the photosynthetic activity of cotton crops facing waterlogged conditions and they have revealed that apart from nutrients inadequacy other factors are also accountable for reduced photosynthesis and leaf necrosis Waterlogging hindered cotton morphological, physiological, and biochemical processes (Armstrong and Drew, 2002). Excess moisture diminishes root growth, reduces H^+^-ATPase activity of plasma membrane, impedes nutrient acquisition, and triggers the activity of 1-aminocyclopropene-1-carboxlic acid in root tissues of cotton (Leblanc et al., 2008).

#### Temperature stress

Temperature is one of the most important factors for agriculture production and is intimately connected (Reddy et al., 2017; Afzal et al., 2020; Ali et al., 2021; Haider et al., 2021). The temperature fluctuates or varies spatially and temporally over the cotton cultivating regions. The growth and productivity of cotton are tremendously decreased at low temperatures, especially during seed germination and crop emergence (Ashraf, 2002). Cotton crops experienced high temperatures lead to reducing the concentration of nutrients in different plant parts, though impacts may vary among nutrients and crop species (Shakoor et al., 2017). High temperature retards the production of antioxidants which accelerate the genesis of reactive oxygen species and thus imparts oxidative stress and nutrient deficiency in cotton (Sarwar et al., 2017). The activity of nitrate reductase involved in nutrient metabolism is also significantly diminished during heat stress (Hungria and Kaschuk, 2014). Under heat stress root density, root mass, and root volume is impaired which ultimately led to a reduction in total nutrient uptake (Basirirad, 2000).

#### Light stress

Sunlight is an indispensable factor for photosynthesis in crop plants. Inadequacy of sunlight leads to impairment in the growth and development of crop plants. Cotton canopy experiencing low light has lower photosynthetic saturation compared to cotton canopy getting optimum light (Landivar et al., 2010). Very less information is available on the impact of light on nutrient uptake in cotton. However, some past studies have reported that total N P, K, S, Ca, and Mg concentrations in the cotton leaf blades increased by 19%, 29%, 22%, 22%, 13%, and 16%, respectively, in shaded plants compared with those of receiving optimum light (Zhao and Oosterhuis, 1998). The impact of different abiotic stresses on cotton morphological and yield attributes are presented in Table 1.

**Table 1:** Impact of different abiotic stresses on the morpho-physiological traits and yield of cotton.

Type of stress	Treatments	Effect/s	References
Salinity stress	Irrigation with saline water at different salinity level	Caused a 50% yield reduction at 17 dS m^−1^	Ahmad et al. (2002) and Khorsandi and Anagholi (2009)
	Cultivation of crops on low (1.15 dS m^−1^), medium (6.0 dS m^−1^), and high salinity levels (11.6 dS m^−1^)	Fibre cellulose content reduced by 17%–42% at high soil salinity level	Peng et al. (2016)
High temperature stress	Cultivation of cotton at 30°C, 35°C, and 40°C	Retention of cotton bolls decreased by 100% at 40°C due to enhanced abortion of squares and young bolls	Hodges et al. (1993) and Oosterhuis and Snider (2011)
	Growing of cotton at different day/night temperature regimes	Cotton plants when exposed to 36/28°C (day/night) temperature, retained 70% less bolls than plants grown under 30/22°C (day/night) temperature	Zhao et al. (2005)
Waterlogging stress	Exposure of waterlogging at four growth stages of cotton	Waterlogging reduced cotton yield by 38.8%, 27.9%, 18.3%, and 7.6% at flowering, squaring, seedling, and boll opening stage, respectively	Wang et al. (2017)
	Waterlogging stress at reproductive stage of crop	Malondialdehyde (MDA) content in cotton roots were increased by 12.8%–93.1% after 8 days of waterlogging at the flowering and boll-setting stages. Since MDA is a major indicator of cell membrane damage	Guo et al. (2010)
Drought stress	Drought stress imposed by skipping or reducing the number of irrigation (0, 1 and 2)	No irrigation caused a 55.3% and 44.7% yield reduction in Sahel and Mehr cultivars	Alishah and Ahmadikhah (2009)
	Maintained three soil water levels (75 ± 5, 60 ± 5, and 45 ± 5% of field capacity) during boll development to determine the influence of drought on yield distribution and fibre quality	Drought reduced the lint yield by 31–35 and 57–60% under 60 ± 5% and 45 ± 5% field capacity, respectively	Wang et al. (2016)
Shading/light stress	Three shading treatments were imposed, which included a non-shaded control (CRLR, crop relative light rate; 100%), mild shading (CRLR 80%), and severe shading (CRLR 60%)	These treatments decreased boll number, boll weight and lint yield by 9%–16%, 0.1%–11% and 10%–27%	Liu et al. (2015)
	Two different mesh of white polyethylene screens were placed over the cotton canopy and were suspended 2.0 m above the soil to provide two shading treatments	Shading reduced lint yield by 17%–38%	Lv et al. (2013)

## Advances in nitrogen utilization in cotton plants and factors involved

### Response in phonemics

**Figure 2:**
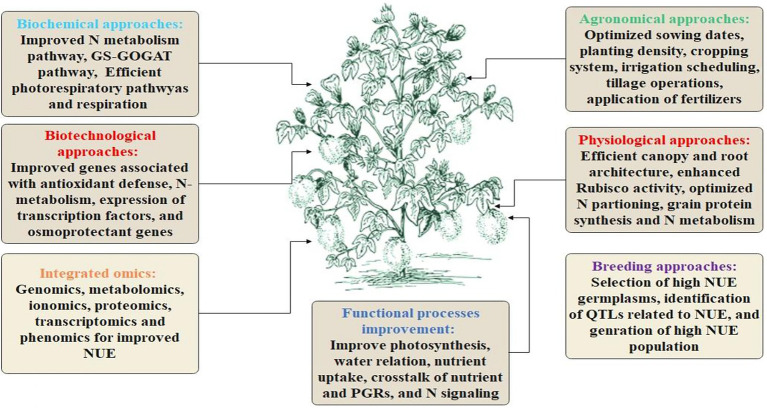
Highlights the physiological, biochemical, biotechnological, agronomical, breeding and omics approaches for enhancing NUE in cotton.

Traditional phenotyping is time-consuming, labor-intensive, expensive, and provides low-throughput and non-reliable phenotypes, impeding cotton breeding (Furbank and Tester, 2011). However, the modern day’s automated phenomic approaches are precise, time-saving, and high-throughput and can circumvent the limitations of conventional breeding. These approaches are being utilized for the effective identification of high-yielding cultivars with additional superior qualities that might be used in the genetic modification of cotton. The advanced breeding, biotechnological, physiological, agronomical, and omics approaches for NUE enhancement in the cotton crop are highlighted in Figure 2.

#### Cotton plant statue

Understanding the exact plant architecture is very crucial for improving cotton yield. For example, Paproki et al. (2011) applied a top-down approach for smart segmentation of cotton for automated analysis of phenotypic data to understand the architecture of the plant and further improvement of the 3D plant reconstruction in cotton. However, as cotton plants differ in shape, no single method is robust enough to offer correct segmentation in all circumstances.

#### Cotton growth and development

Plant productivity may well be determined by understanding of plant growth dynamics. Plant growth modeling and functional analysis using a high-throughput phenotyping platform give a better understanding of energy distribution in the plant. Jiang et al. (2018) developed the ‘GPhenoVision’ system for evaluating the usefulness of the field-based high throughput phenotyping using plant growth and fiber yield traits for futuristic breeding programs and genomics studies in cotton. Another high throughput terrestrial LiDAR-based phenotyping system was developed by Sun et al. (2018) for accurate screening and understanding of growth trends and growth rate based on canopy height, projected canopy area, and plant volume of cotton plants through 3D surface modeling. Recently, CSM-CROPGRO model was used in cotton to evaluate the growth and development of the plant under varying nitrogen doses and planting dates (Ur Rahman et al., 2019).

#### Cotton yield and its components

Phenomic approaches have been used in several crops for precise identification of phenotypes with high agronomic and economic importance by using advanced crop sensing platforms and data analysis software. Thorp et al. (2015) investigated proximal hyperspectral sensing with a field for estimating leaf water content, specific leaf mass, leaf chlorophyll content, and leaf area index which could be utilized in cotton improvement. Phenomics data in association with a genome-wide association study (GWAS) on 56 morphological and 63 texture traits identified the 390 genetic loci in cotton (Li et al., 2020).

#### Cotton plant adaption to stress

**Figure 3:**
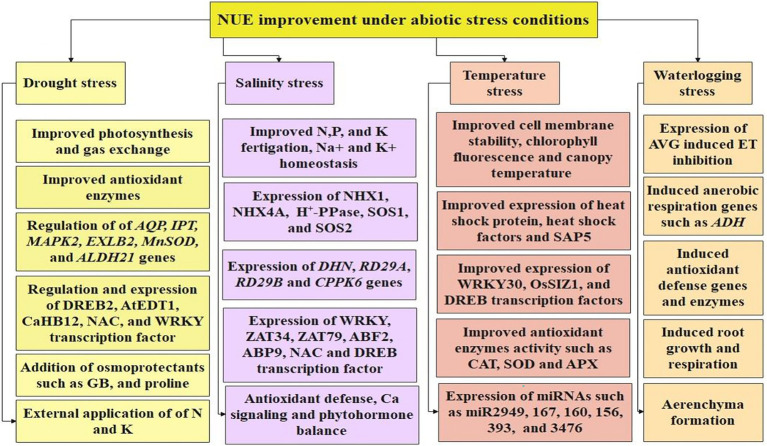
Highlights the role of different transcription factors, genes, and functional traits in enhancing tolerance to drought, salinity, temperature, and waterlogging stresses.

Adaptation to environmental stresses by a cotton plant directly determines its growth and yield. Understanding the accurate phenotypic and genotypic relationship makes it easier identification of stress-tolerant lines. Drought resistance is a complex trait governed by multiple genes. Li et al. (2020) through phenomics (using novel image-based indicators) and genomics (using Genome-Wide Association Studies) approach identified *Gh_A04G0377* and *Gh_A04G0378* candidate genes negatively regulating the drought resistance in cotton. The physiological, biochemical, and molecular mechanisms related to abiotic stress tolerance in cotton are highlighted in Figure 3.

### Response in metabonomics

#### N related metabolism

Since nitrogen is a key ingredient of macromolecules such as proteins, enzymes, metabolites, signaling molecules, and plant hormones, it is one of the most significant factors limiting global cotton production. Metabolomics sheds light on the fundamental mechanisms of nitrogen absorption and assimilation in the plant, which ultimately determine its growth performance and productivity. Iqbal et al. (2020) through metabolic and transcriptomic approaches elucidated the major biosynthetic pathways and transcripts involved in Carbon and Nitrogen metabolism in cotton. Increased accumulation of 2-ketoglutarate, succinate, malate, etc. in the *Ligon lintless-2* cotton mutant also indicated a higher nitrogen compound metabolism (Naoumkina et al., 2013).

#### Carbon related metabolism

N assimilation is associated with carbon metabolism and subsequent biomass yield. Carbon is metabolized into cellulose fibers in cotton. The first comprehensive study on changes in metabolites and associated transcripts involved in elongating fibers in *Ligon lintless-2* mutant was reported by Naoumkina et al. (2013). They highlighted the number of factors and metabolic mechanisms involved in cotton fiber elongation. Similarly, Tuttle et al. (2015) have given a deep insight of cell wall transitions to secondary wall synthesis using deep-sequencing transcriptomics and non-targeted metabolomics approaches. They identified 206 fiber metabolites and 38,000 fiber-related transcripts and implicated that lignification is transcriptionally repressed during secondary cell wall synthesis in cotton. The roles of sterol and sphingolipid derivatives in the initiation of fiber cells have been identified through comparative metabolomics analysis in cotton (Wang et al., 2021a,b,c,d). Brown cotton fiber is a one-of-a-kind raw material made from naturally pigmented cotton. In a recent study, Wang et al. (2021a,b,c,d) elucidated the transcriptional regulatory network of proanthocyanidins biosynthesis and flavonoid metabolism in brown fiber variety (Zong 1–61) and white near-isogenic lines (RT) of cotton. They detected 10,891 differentially expressed genes about the brown pigmentation in cotton.

#### Stress-related metabolism

Various studies have reported stress-related metabolism in cotton crops. For example, Khizar et al. (2020) identified 241 *Aspergillus* leaf spot resistance-related metabolites belonging to phenylpropanoids, flavonoids, alkaloids, terpenoids, etc. through non-targeted metabolomics in cotton. Cotton yield is influenced by abiotic stress, particularly drought stress. Recently Zhang et al. (2021) identified a total of 537 metabolites (ABA, amino acids, Flavonoids, ROS, etc.) were detected at different stages of cotton cultivars, i.e., XLZ8 (sensitive) and G95079 (tolerant) under drought stress.

### Response in transcriptome

The assimilation of nitrogen is generally associated with photosynthetic efficiency and carbon content in the plant which is controlled by the mechanism involved in carbon-nitrogen balance (Ranathunge et al., 2014). This carbon-nitrogen ratio plays a crucial role in the proper growth and development of plants (Zheng, 2009). Various reports have demonstrated that the well-organized systemic pathways are involved in C and N metabolism that have a major impact on the growth yield and productivity of plants (Krapp and Traong, 2006). In this review information regarding gene regulation for N metabolism and its effect on plant growth and development, yield, productivity, and C and N metabolism has been taken into consideration (Li et al., 2016a,b). Furthermore, the carbon and nitrogen ratio plays a pivotal role in improving NUE, as it is essential for a plant to assimilate maximum carbon which enhances the plant’s capacity for nitrogen uptake and utilization. Similarly N content in plants can have a major impact on the carbon fixation metabolism of plants (Li et al., 2017), as N is required by plant in ample amount for production of photosynthetic enzymes, for example, Rubisco and PEP carboxylase. Decreased N accumulation and storage results in low carbon fixation in a plant (Li et al., 2017). Besides the carbon to nitrogen ratio, the outcome products of the GS and GOGAT cycles are also important for plants. Glutamate helps in nitrogen transportation, acts as a singling molecule, and also it provides a substrate for the synthesis of amino acids (Inthapanya et al., 2000). It also controls and regulates C and N pathways and ratios in plants. Enhanced expression of PEP carboxylase in plants resulted in increased glutamine synthesis (Inthapanya et al., 2000). The alterations in the relationship of source or sink in plant coordinated with proportionate changes in carbon or nitrogen ratio must be associated with environmental stimulants which include factors like nitrogen availability (Harvey, 1939). Although C-to-N ratios are responsible for proper plant growth and development, it also acts as a limiting factor while examining Nitrogen use efficiency in plants (Koutroubas and Ntanos, 2003). Nitrogen uptake, accumulation, and remobilization are greatly influenced by carbon metabolism, thus affecting NUE unless there is an increase in carbon content which is also confirmed by RNA sequencing studies conducted in cotton.

Furthermore, significant alterations were also observed in the metabolic pathways of different amino acids which include phenylalanine, tryptophan, tyrosine cysteine, methionine metabolism, alanine, glutamate, aspartate, glycine, serine and threonine, and “starch and sucrose metabolism” similarly glycolysis – gluconeogenesis also acts as an important factor in carbon metabolism which was further restored and was highly expressed in the shoot of CCRI-69 when N was supplied after a certain interval of time within 0–6 h. Furthermore, 4 DEGs associated with pentose phosphate shunt were highly expressed in the shoot of CCRI-69.

### Potential candidate genes that can be utilized for transgenic development in cotton for high nitrogen use efficiency

NUE amelioration is considered to be a crucial challenge in a cotton crop that has to be overcome. Plenty of research work has been done to improve cotton NUE that mainly focused on crop cultivation techniques (Zhang et al., 2018) and now it has been directed towards NUE evaluation of cotton varieties and their application in breeding programs (Iqbal et al., 2020). These strategies adopted to increase NUE in the cotton crop are time-consuming, laborious, and costly so alternative approaches are being utilized. Therefore, the transgenic methodology is now preferred to enhance agronomic traits and has received great success in cotton. The success of *Bt* cotton for insect resistance (Zafar et al., 2020) is a great example of a transgenic crop. Therefore, determination and genetic alteration of major NUE-efficient genes become a crucial step towards improving cotton NUE.

It has been reported by experiment that *Arabidopsis NLP7* gene integration in cotton helps to increase NUE. The *AtNLP7* over-expressed genes lines in cotton exhibited significantly better growth in seedlings under different N levels with improved seedling biomass, wider leaf, and healthy roots, which was directly useful during the seedling establishment process. The field trial indicated that higher expression of *AtNLP7* greatly improved both cotton NUE and yield. It was also observed that *AtNLP7* genes are also related to N uptake and assimilation in cotton. Its integration into the genome of cotton resulted in increased uptake of N uptake and a higher assimilation rate therefore useful in imparting cotton with high NUE and a better yield (Jan et al., 2022).

Several studies provide evidence that there are in general 60, 61, and 105 *NLP* genes in *G. arboreum*, *G. raimondii*, and *G. hirsutum*, approximately (Magwanga et al., 2019). BLAST results were evident to prove that there are 7 orthologs of *AtNLP7* that are found in cotton. A list of these important 7 genes includes *GhNLP2, 4, 5, 6, 7, 8, and GhNLP9*. furthermore, the NREs sequence in the promoter (2 kb) of these cotton genes is identical to those observed in the case of *Arabidopsis* which indicates that AtNLP7 could be responsible for activating the up-regulation of cotton genes by associating with NRE. Similarly, various regulatory sequences have been discovered that are useful in improving NUE, e.g., *SNF1*-related kinase in tomato (Wang et al., 2012), *bHLH T.F* in soybean (Chiasson et al., 2014), *NAC T.F* present in wheat (Borrill et al., 2017). These genes can be successfully used to develop transgenic cotton with enhanced NUE.

Fixation of N and C are regulated in association, which is the indication that NUE, along with N accumulations, also affects C metabolism (Chardon et al., 2012). Thus, therefore it is evident enough to prove that optimization in plant growth, development, and yield includes concurrent amelioration in both N and C use efficiencies. It was observed in research conducted by Yu et al., 2016 that association among C and N accumulation resulted in higher storage of C and N in transgenic cotton lines where *AtNLP7* gene was highly expressed. The high expression level of *AtNLP7* resulted in higher chlorophyll in transgenic cotton which may have a direct effect on the C-fixation ability of cotton (Yu et al., 2016). A proper N/C ratio is highly useful in increasing NUE.

The *AtNLP7-increased* NUE in the cotton crop was further assisted by the data of total N and C content and two biochemical metabolite markers chiefly glutamate and glutamine for nitrogen accumulation and utilization. High levels of nitrogen and carbon content were observed in transgenic cotton as compared with non-transgenic cotton. The net glutamate contents of both non-transgenic and transgenic plants were higher at a higher level of KNO_3_. On the other hand, glutamine (Gln) contents increased exceptionally in transgenic cotton than in control while net Gln quantity increased on high supply of N as compared with lower quantity of N. These results show that cotton having enhanced expression of *AtNLP7* gene will improve nitrogen use efficiency in cotton and provide an alternative approach as compared to breeding and agronomic methodologies (Jan et al., 2022).

These data can support by the expression studies of nitrogen metabolism marker genes which include *GhNRT1.1*, *GhNRT1.2*, *GhNRT2.1*, *GhNRT2.2*, *GhNIA1*, *GhAMT1*, *GhAMT2*, and *GhGST1.1,* which were expressed in transgenic lines shows that the activity of AtNLP7 gene is conserved in cotton crop (Jan et al., 2022). Some genes related to NUE in cotton plants are highlighted in Table 2.

**Table 2:** Some genes and transporters related nitrogen use efficiency (NUE) in cotton plants.

Genes/transporters	Associated with/functions	References
*AtNLP7*	Improved N uptake, its assimilation, lint yield and NUE	Jan et al. (2022)
*AtNLLP7*	Improved N assimilation, yield and NUE	Jan et al. (2022)
*GhNRT1.1, GhNRT1.2, GhNRT2.1, GhNRT2.2*	As nitrate transporters improved NUE	Jan et al. (2022)
*GhNIA1, GhGST1.1*	As nitrate transporters improved NUE	Jan et al. (2022)
*GhAMT1, GhAMT2*	As ammonium transporters improved NUE	Jan et al. (2022)
*GhGS1*	Glutamine synthetase/NUE	Jan et al. (2022)
*Ghir_A09G009680, Ghir_D09G009410, Ghir_D05G008910, Ghir_A05G008920, Ghir_A09G009700, Ghir_A12G008140, Ghir_D12G007600, Ghir_A12G008140, Ghir_D07G011260, Ghir_A05G008930,* *Ghir_A12G008140, Ghir_A05G008930,* *Ghir_D09G009430, Ghir_A12G004130, Ghir_A12G008140, Ghir_D13G010010, Ghir_A12G008140, Ghir_D09G009430*	Vegetative and reproductive organs, fiber development, and N metabolism	Iqbal et al. (2022)
*Ghir_D09G020360, Ghir_A09G009680,* *Ghir_A13G023660, Ghir_A07G018790,* *Ghir_A09G020960, Ghir_D13G024390,* *Ghir_D12G026390, Ghir_D02G003120,* *Ghir_A02G002720, Ghir_A13G005930,* *Ghir_D09G020360, Ghir_A13G023660,* *Ghir_A09G009700, Ghir_D13G024390*	Involved in N metabolism and improved NUE	Iqbal et al. (2020)
*Gh_A05G3286 (NLP5)*	Enhanced plant tolerance to N-limited stress	Magwanga et al. (2019)
*AMT1;1, gdh3 and gdh2*	Involved in N assimilation, improved seedling growth under limiting N conditions	Feng et al. (2020)
*GhAMT1.3*	Facilitated NH^4+^ acquisition and utilization	Sun et al. (2018)
*OsARG*	Influenced the crop morphology and N transition of seedlings	Meng et al. (2015)

## Conclusion

**Figure 4:**
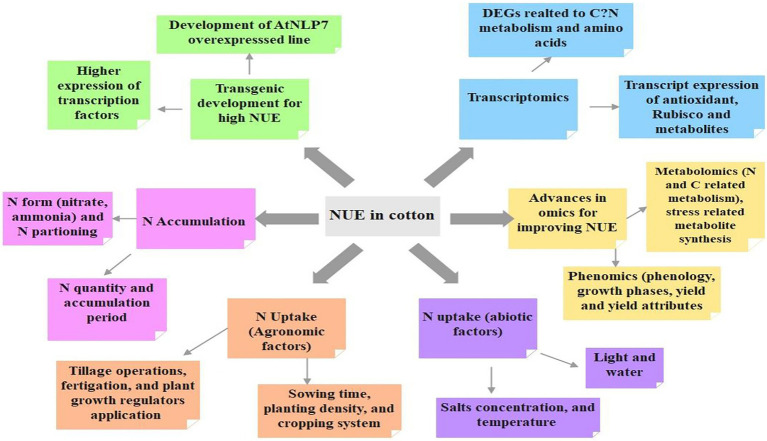
Highlights the multidimensional approaches (agronomical, biochemical and molecular) to enhance NUE in cotton plant.

Cotton is a major source of high-quality natural fiber for textile industries globally however, its production is directly associated with the NUE, which relies on the management practices like source, rate, time, and mode of N administration, growth stage, and genotypic constitution. Excess N dose reduction, timely application, and gradual and controlled N release may be beneficial for improved NUE in cotton. A mulidimension approach to enhance NUE in cotton crop highlighted in Figure 4. Further, the usage of N efficient genotypes can enhance NUE and cotton yield while reducing the possibility of excess N wastage, and environmental pollution simultaneously. Furthermore, overexpression of various target genes like *Arabidopsis NLP, NLP7,* and *AtNLP7* can be extremely useful in increasing NUE, plant growth, and fiber yield in cotton even under poor N availability. Although several conventional studies on manipulating single or multiple genes to improve NUE have been reported in a variety of crops, the use of modern days advanced genomic tools like whole genome sequencing and omics could be the thrust areas for futuristic research in cotton. In addition, the use of wild genotypes may be an appropriate alternative to improve NUE by retaining the resistance against major pests and diseases in cotton.

## Author contributions

ANS, TJ, RKS, RS, DW, SH, HA, DJ, HP, NRA, RYG, and MJ together designed the scope of the manuscript, wrote individual chapters, and prepared the figures. All authors contributed to the article and approved the submitted version.

## Funding

The authors thank Smart Health Initiative and Red Sea Research Center, Division of Biological and Environmental Sciences and Engineering, King Abdullah University of Science and Technology, Thuwal, Saudi Arabia for financially supporting the current research. The authors also extend their appreciation to Alexandria University for supporting the current research.

## Conflict of interest

The authors declare that the research was conducted in the absence of any commercial or financial relationships that could be construed as a potential conflict of interest.

## Publisher’s note

All claims expressed in this article are solely those of the authors and do not necessarily represent those of their affiliated organizations, or those of the publisher, the editors and the reviewers. Any product that may be evaluated in this article, or claim that may be made by its manufacturer, is not guaranteed or endorsed by the publisher.
